# The Effect of Different Doses of Zearalenone in Feed on the Bioavailability of Zearalenone and Alpha-Zearalenol, and the Concentrations of Estradiol and Testosterone in the Peripheral Blood of Pre-Pubertal Gilts

**DOI:** 10.3390/toxins12030144

**Published:** 2020-02-26

**Authors:** Łukasz Zielonka, Magdalena Gajęcka, Sylwia Lisieska-Żołnierczyk, Michał Dąbrowski, Maciej T. Gajęcki

**Affiliations:** 1 Department of Veterinary Prevention and Feed Hygiene, Faculty of Veterinary Medicine, University of Warmia and Mazury in Olsztyn, Oczapowskiego 13/29, 10-718 Olsztyn, Poland; lukaszz@uwm.edu.pl (Ł.Z.); michal.dabrowski@uwm.edu.pl (M.D.); gajecki@uwm.edu.pl (M.T.G.); 2 Independent Public Health Care Center of the Ministry of the Interior and Administration, and the Warmia and Mazury Oncology Center in Olsztyn, Wojska Polskiego 37, 10-228 Olsztyn, Poland

**Keywords:** zearalenone, bioavailability, estradiol, testosterone, blood concentration, pre-pubertal gilts

## Abstract

The objective of this study was to determine the effect of long-term (48 days), *per os* administration of specific zearalenone (ZEN) doses (20 and 40 μg ZEN/kg BW in experimental groups EI and EII, which were equivalent to 200% and 400% of the upper range limit of the no-observed-adverse-effect-level (NOAEL), respectively) on the bioavailability of ZEN and the rate of changes in estradiol and testosterone concentrations in the peripheral blood of pre-pubertal gilts. ZEN and α-ZEL levels were similar until day 28. After day 28, α-ZEL concentrations increased significantly in group EI, whereas a significant rise in ZEN levels was noted in group EII. The presence of estradiol in peripheral blood plasma was not observed until day 20 of the experiment. Spontaneous secretion of estradiol was minimal, and it was determined at very low levels of up to 10 pg/mL in EI and EII groups. Testosterone concentrations ranged from 4 to 9 ng/mL in all groups. A decrease in the concentrations of both analyzed hormones was reported in the last stage of the experiment. The results of the experiment indicate that: (i) The bioavailability of ZEN in peripheral blood has low diagnostic value, (ii) exposure to low doses of ZEN induces minor changes in the concentrations of the analyzed hormones, which could lead to situational supraphysiological hormone levels and changes in endogenous hormonal balance.

## 1. Introduction

The majority of environmental estrogens (not all of them have toxic or contaminating effects) are endocrine disrupters (EDs) [1] which are commonly encountered in soil, air, water, food products, and animal feeds [2,3]. Phytoestrogens (e.g., genistein and coumestrol) and mycoestrogens (including ZEN produced by moulds) [4,5] are natural EDs found in the environment.

Zearalenone is a resorcylic acid lactone chemically described as 6-(10-hydroxy-6-oxo-*E*-1-undecenyl)-β-resorcylic acid lactone (C_18_H_22_O_5_, molar mass: 318.36 g/mol, CAS: 17924-92-4). Zearalenone is a white crystalline compound with melting temperature of 164–165 °C. This mycotoxin is insoluble in water, but it is soluble in alkaline solutions and organic solvents. Zearalenone remains stable during storage, grinding and processing, including thermal processing [4].

Endocrine disruptors also influence the transport and distribution [6] of endogenous hormones, leading to changes in their concentrations and free hormone levels [7,8]. The effect of endogenous hormones and xenoestrogens on ion channels is an example of such activity. In general, EDs including environmental mycotoxins, such as ZEN and its metabolites, disrupt the activity of Ca^2+^ channels and/or Ca^2+^ signaling in certain types of cells [9]. These findings indicate that endogenous hormones and EDs exhibit hormonal activity by binding to nuclear hormone receptors. By acting outside the normal hormonal milieu, they are capable of producing unexpected results [10,11,12].

Zearalenone and its metabolite α-zearalenol (α-ZEL) have a similar chemical structure to estrogen, but unlike steroids, they do not originate from sterane structures [6]. Endocrine disruptors, including ZEN, are involved in several mechanisms [7,13] that can influence hormonal systems [14] and produce adverse results, in particular in pubertal gilts: (i) They compete with endogenous estrogens for estrogens receptors (ERs) or androgen receptors (ARs) binding sites, which leads to changes in *m*RNA expression and protein synthesis and reduces the effectiveness of endogenous steroid hormones, and they are responsible for the transport of ZEN inside cells [6,12,15]; (ii) they can bind with the receptor without activating it, and the presence of a substance on the receptor prevents natural hormone binding (antagonistic effect) [11,16]; (iii) they can bind with blood transport proteins to lower the concentrations of natural hormones in blood [17]; (iv) by disrupting metabolic processes in the body, EDs may affect the degree of synthesis/breakdown and the release of natural hormones [6,18,19].

The functions of the endocrine system are modified when physiologically active ligands (hormones) such as estradiol (E_2_) and testosterone (T) are present at low concentrations [20,21]. Endogenous hormones and EDs act via receptors, but their activity is determined by, among others, the applied dose [22]. Lower doses of EDs influence the production, metabolism, absorption, and secretion of hormones because minor changes in hormone concentrations have far-reaching biological consequences [23]. Endogenous hormones are active in blood at very low concentrations for several reasons: (i) Receptors have high affinity for specific hormones, and they are capable of accepting a sufficient number of molecules to produce the most effective response [11]; (ii) a nonlinear correlation exists between hormone concentrations and the number of saturated receptors [22]; (iii) a nonlinear correlation also exists between the number of saturated receptors and the magnitude of a biological effect [7].

The objective of this study was to determine the effect of 48-day administration of ZEN doses of 20 and 40 μg ZEN/kg BW (equivalent to 200% and 400% of the highest NOAEL value, respectively), on the bioavailability of ZEN and the concentrations of E_2_ and T in the peripheral blood of pre-pubertal gilts.

## 2. Results

### 2.1. Bioavailability of ZEN and Its Metabolite

**Figure 1:**
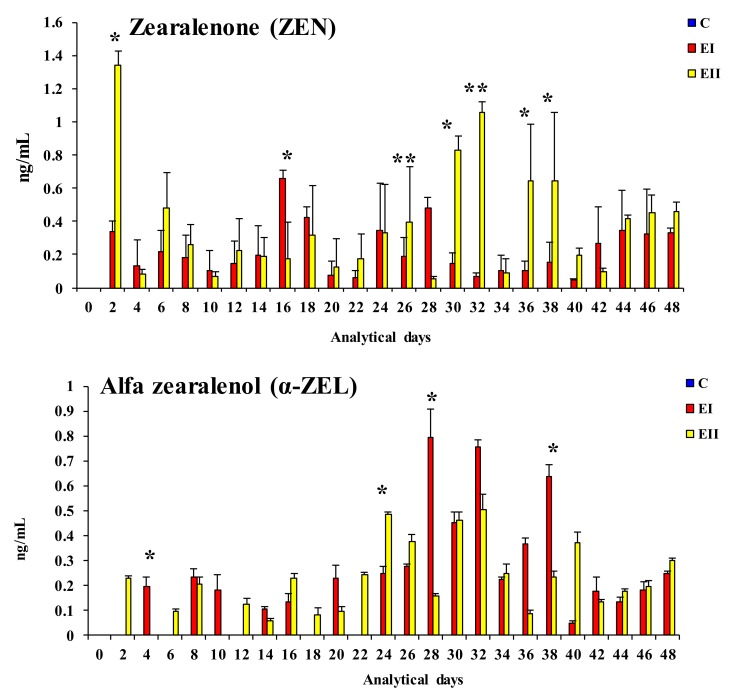
Arithmetic means ($\bar{x}$) and standard deviation (SD) for the bioavailability of zearalenone (ZEN) and α-zearalenol (α-ZEL) (ng/mL) in the peripheral blood of pre-pubertal gilts on different analytical days and in the experimental groups (group EI 20 μg ZEN/kg BW; group EII 40 μg ZEN/kg BW). Limits of detection (LOD) > values below the limit of detection were regarded as equal to 0. Statistically significant differences were determined at * *p* ≤ 0.05 and ** *p* ≤ 0.01.

The blood plasma bioavailability [24] of ZEN and α-ZEL on different days of the experiment is given in Figure 1. ZEN and its metabolite were determined in both experimental groups. Their presence was not observed in control group animals (values below limits of detection—LOD—regarded as equal to 0).

**Figure 2:**
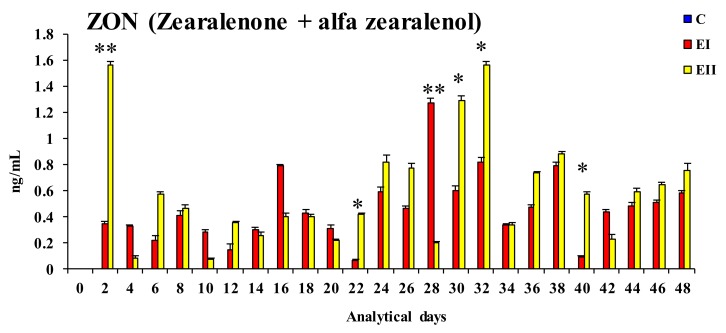
Arithmetic means ($\bar{x}$) and standard deviation (SD) for the bioavailability of ZON (ZEN + α-ZEL) (ng/mL) in the peripheral blood of pre-pubertal gilts on different sampling dates and in the experimental groups (group EI 20 μg ZEN/kg BW; group EII 40 μg ZEN/kg BW). Limits of detection (LOD) > values below the limit of detection were regarded as equal to 0. Statistically significant differences were determined at * *p* ≤ 0.05 and ** *p* ≤ 0.01.

Significant differences (* *p* < 0.05) were observed between groups EII and EI: (i) In the bioavailability of ZEN on days 2, 16, 30, 36, and 38 and in their mean values for the entire experimental period (EI < EII on all sampling dates, excluding day 16); (ii) in the bioavailability of α-ZEL on days 4, 24, 28, and 38 (EI < EII on the first two sampling dates, and EI > EII on the remaining days); (iii) in the bioavailability of ZON (see Figure 2) on days 22, 30, 32, and 40, and in their mean values during the entire experiment (EI < EII on all sampling dates). Highly significant differences (** *p* < 0.01) were noted between groups EI and EII: (i) In the bioavailability of ZEN on days 28 (EI > EII) and 32 (EI < EII) and in ZON concentrations on days 2 (EI < EII) and 28 (EI > EII).

### 2.2. Estradiol Concentrations

**Figure 3:**
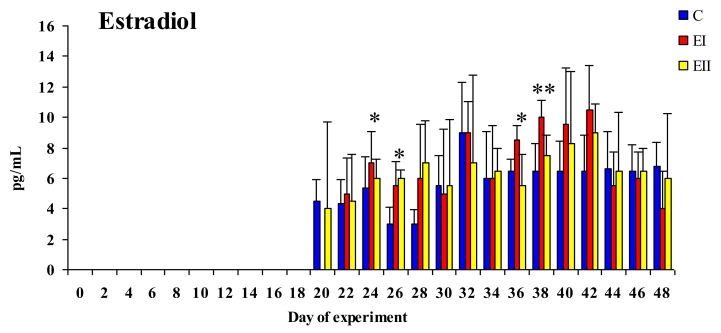
Arithmetic means ($\bar{x}$) and standard deviation (SD) for the concentrations of estradiol (pg/mL) in the peripheral blood of pre-pubertal gilts on different sampling dates and in the experimental groups (group EI 20 μg ZEN/kg BW; group EII 40 μg ZEN/kg BW). Limits of detection (LOD) > values below the limit of detection were regarded as equal to 0. Statistically significant differences were determined at * *p* ≤ 0.05 and ** *p* ≤ 0.01.

On the first ten sampling dates, the concentrations of E_2_ were below the limit of sensitivity in all experimental groups (see Figure 3). This was also noted in group EI on day 20. Significant differences at *p* < 0.05 were noted between groups EI and C on days 24 and 36 (EI > C), and between groups EII and C on day 26 (EII > C). Highly significant differences at *p* < 0.01 were observed between groups EI and C on day 38 (EI > C).

Between experimental days 36 and 42, a clear increase in E_2_ levels was noted in both E groups in comparison with group C (see Figure 3). The concentrations of E_2_ in the analyzed pre-pubertal gilts ranged from 3.0 to 10.5 pg/mL during the entire experiment. The mean increase in the values of E_2_ was similar during the experiment, but considerably higher in both E groups until day 42. The concentrations of E_2_ in experimental groups decreased towards the end of the experiment in comparison with control. The mean values of E_2_ in peripheral blood were determined at 3.46 pg/mL in group C, 3.90 pg/mL in group EI and 3.83 pg/mL in group EII on 24 sampling dates, and at 5.77 pg/mL, 6.33 pg/mL, and 6.22 pg/mL, respectively, on the last 15 sampling dates (see Figure 3).

### 2.3. Testosterone Concentrations

**Figure 4:**
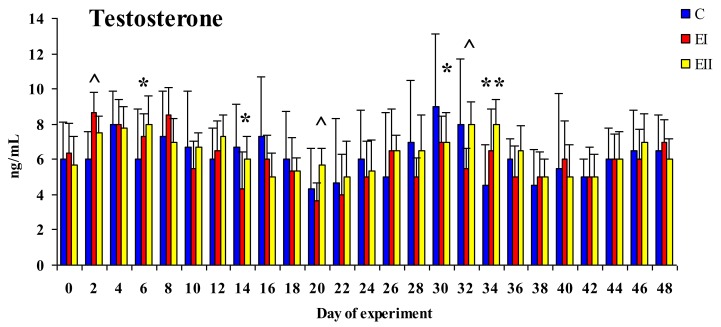
Arithmetic means ($\bar{x}$) and standard deviation (SD) for the concentrations of testosterone (ng/mL) in the peripheral blood of pre-pubertal gilts on different sampling dates and in the experimental groups (group EI 20 μg ZEN/kg BW; group EII 40 μg ZEN/kg BW). Statistically significant differences were determined at * or ^ *p* ≤ 0.05 and ** *p* ≤ 0.01; *—EI or EII compared with C; ^—EII compared with EI.

Testosterone concentrations in blood plasma samples from pre-pubertal gilts are presented in Figure 4. During the entire experiment, T concentrations in the analyzed pre-pubertal gilts ranged from 4.0 to 9.0 ng/mL. Testosterone concentrations were higher in group C on 15 out of 25 sampling dates (60%). The changes in T concentrations were highly similar during the experiment. The mean concentration of T in peripheral blood during the entire experiment (25 sampling dates) was determined at 6.18 ng/mL in group C, 5.98 ng/mL in group EI, and 6.35 ng/mL in group EII.

Statistically significant differences at *p* < 0.05 were reported between groups EI and C on days 14 and 30 (C > EI), between groups EII and C on days 6 (C < EII) and 30 (C > EII), and between groups EII and EI on days 2 (EI > EII), 20 (EI < EII), and 32 (EI < EII). Significant differences at *p* < 0.01 were observed between groups EII and C on day 34 (C < EII). The differences were statistically significant on days 6 and 34. A decrease in T concentrations in the experimental groups, relative to the control group, or no changes in T levels were observed more frequently.

## 3. Discussion

According to Shephard [25], animals are constantly exposed to mycotoxins which are secondary products of mould metabolism. Their elimination depends on the animal’s age and the bioavailability of toxins in the blood plasma. This suggests that mycotoxins are always present in the natural environment, but at LOD doses. The bioavailability of ZEN and α-ZEL in the blood plasma is determined by the rate and the form of their biotransformation to water-soluble substances which are broken down by enzymes in the liver or other tissues as part of the detoxification process. According to some authors [26,27], biotransformation can generate products which are more toxic (bioactivation) than the parent mycotoxin. One of such compounds is ZEN which is transformed to α-ZEL in pre-pubertal gilts, and the consequences of this process are visible in tissues sensitive to that metabolite [13]. Tiemann et al. [28] observed that a decrease in steroid synthesis in cultures of porcine ovarian follicular granulosa cells resulting from the inhibition of P450scc activity is inversely proportional to the α-ZEL dose.

### 3.1. Zearalenone and Its Metabolite

In this study, the bioavailability of ZEN and α-ZEL in the blood plasma indicates that ZEN levels remained low in the peripheral blood of the experimental pre-pubertal gilts (groups EI and EII) in the first 28 days of the experiment. This can probably be attributed to enhanced biotransformation of ZEN and the distribution (carryover) of ZEN and α-ZEL to estrogen-sensitive tissues such as enterocytes, in particular in the duodenum and jejunum [19] in the initial period of exposure [29] and, subsequently, their bioavailability in peripheral blood.

**Figure 5:**
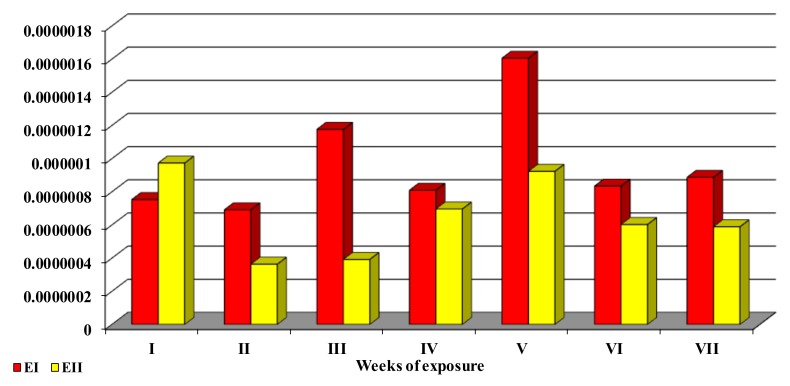
The bioavailability factor. The mean values ($\bar{x}$) of the ratio of ZON (ZEN + α-ZEL) bioavailability in peripheral blood (ppb) to ZEN concentration in the diet (ppb), expressed as the total dose administered in each week of the experiment.

The decrease in the values of ZEN in peripheral blood (see Figure 5) in both groups in the first two weeks of exposure provides evidence for the above, and it could indicate that: (i) All forms of mycoestrogen are intercepted by enterocytes (at absorptive level) or (ii) estrogen-sensitive tissues have a high demand for estrogen (at post-absorptive level) in pre-pubertal animals. In this experiment, the bioavailability of ZEN in peripheral blood 30 min after *per os* administration (maximum ZEN concentration in the blood [11]), was very low in comparison with the values reported by Dänicke and Winkler [30] and the values noted in intestinal tissues by Zielonka et al. [19] and Zheng et al. [31], which makes the resulting data difficult to interpret. It should also be noted that those values are inversely proportional to the ZEN dose (see Figure 5). Therefore, exposure to a lower ZEN dose leads to a hormetic dose response [32] by influencing the concentrations of the parent compound and its metabolite in peripheral blood, as well as the synthesis and secretion of sex steroid hormones (see Figure 3 and Figure 4) [31,33,34]. The above state limits bioavailability. The analyzed values reflect on liver metabolism, i.e., the unmetabolised portion of the active substance (e.g., ZEN) and the produced and unutilised α-ZEL metabolite. The resulting “free ZEN” can be utilized for specific purposes [17], for example, for competing with endogenous E_2_ or coregulating its supply. The above is illustrated by the fact that both doses lead to a significant increase in the concentrations that induce hyperestrogenism although rather supraphysiological hormone levels (increase in the concentrations of E_2_ in both E groups and increase in T levels in group EII relative to group C) [34,35]. This observation points to a counter response to the presence of specific ZEN doses in pre-pubertal gilts. The presented results can be extrapolated [19,33,34] to suggest that “free ZEN” is captured by estrogen receptors (ERs) in the digestive tract [36], and that it stimulates qualitative changes (activation?) of ERs. The analyzed scenario testifies to the multidirectional effects of ZEN and its metabolite [33]. Therefore, it could be postulated that a dose of 20 μg ZEN/kg is more effectively utilized by pre-pubertal gilts. The above implies that biotransformation processes proceed in an identical manner, but the bioavailability of ZEN is higher in group EII and the bioavailability of α-ZEL is higher in group EI, whereas the parent compound (ZEN) and its metabolite, α-ZEL, are utilized more effectively in group EI (due to lower supply).

The values of ZON (see Figure 2), i.e., the total concentration of the parent compound and its metabolite, confirm the above suggestions. The values of ZON were higher in group EII on 15 sampling dates (60%). These findings indicate that the bioavailability of ZEN was higher in group EII, but they do not prove that ZEN was more effectively utilized for physiological processes. Low values of ZON at the beginning of the experiment can probably be attributed to the “saturation” of digestive tract tissues, in particular in regions with a higher concentration of ERs [19,36], and the low concentrations of endogenous hormones in animals during sexual maturation [5,8,34,37]. In other words, the involvement of the analyzed hormones in the stimulation of steroidogenesis and the conversion of T to E_2_ ultimately led to supraphysiological hormone levels, in particular in group EII.

### 3.2. Concentrations of Estradiol and Testosterone

Zearalenone is an ED [22], but its presence in the diet of experimental animals did not affect the production of endogenous hormones such as E_2_ and T relative to the control group [38]. The presence of E_2_ (see Figure 3) was not observed on the first nine sampling dates (19 days) of the experiment. On successive sampling dates (15 dates, 53.33%), E_2_ values were higher in both E groups than in group C. The concentrations of T (see Figure 4) were lower on 60% sampling dates in both experimental groups than in the control group, which provides indirect evidence for the acceleration of metabolic processes or the conversion of T to E_2_ [39] under the suppressive influence of ZEN [34,40,41,42]. The concentrations of E_2_ after 20 days of exposure and serum T levels throughout the experiment were directly proportional to the ZEN dose. According to Vandenberg et al. [22], ZEN and/or α-ZEL exert a “complementary” effect on the concentrations of endogenous hormones (see Figure 3 and Figure 4). The above is reflected by an increase in E_2_ levels from day 20 to day 42 of the experiment and the decreasing trend in the concentrations of T between day 2 and the end of the experiment, which could alleviate disruptions in hormonal homeostasis [40,41]. The observed T values could also be attributed to the fact that T regulates sexual differentiation, muscle and bone mass, and erythropoietic and metabolic processes which play a very important role in the development of pre-pubertal gilts. These findings suggest that the physiological demands of young gilts considerably exceed the supply of endogenous E_2_ and T. “Free ZEN” compensates for (or relieves) the resulting endocrinological dysfunction, e.g., by changing the expression of HSD enzymes [34].

### 3.3. Conclusions

The results of endocrinological analyses of selected estrogens (E_2_) and androgens (T) in pre-pubertal gilts, administered at specific doses (200% and 400% of the highest NOAEL value) of ZEN (20 or 40 μg ZEN/kg BW) for 48 days suggest that: (i) The bioavailability of ZEN in peripheral blood is very low and highly varied throughout exposure, and that its diagnostic values are difficult to determine; (ii) experimentally-induced hyperestrogenism or, in other words, “supraphysiological hormone levels” contributed to a minor increase in total concentrations of E_2_ (which could intensify proliferative processes) and influenced the levels of T (T values were lower in the experimental groups on 60% of sampling dates); (iii) the presented results can be extrapolated to suggest that the investigated ZEN doses caused different responses—the lower dose probably generated stimulatory/adaptive effects, whereas the higher dose probably inhibited life processes in the studied animals.

By extrapolation [34], it can be stated that the analyzed ZEN values affect the levels of E_2_ and T in pre-pubertal gilts, thus reducing the risk of fighting for establishing the dominance hierarchy in the herd, contributing to changes in animal behaviour, slowing down sexual maturation, and boosting metabolism. Such observations could be important for pig breeders and veterinarians supervising pig farming and commercial feed production.

## 4. Materials and Methods

All of the experimental procedures involving animals were carried out in compliance with Polish legal regulations determining the terms and methods for performing experiments on animals (Opinion No. 14/2005/N of the Local Ethics Committee for Animal Experimentation at the University of Warmia and Mazury in Olsztyn, Poland in of 26 January, 2005).

### 4.1. Experimental Animals

This study was conducted at the Department of Veterinary Prevention and Feed Hygiene, Faculty of Veterinary Medicine, University of Warmia and Mazury in Olsztyn, Poland, on 18 clinically healthy, two-month-old pre-pubertal gilts. The mean initial body weight was 30 ± 2 kg. The pre-pubertal gilts were housed in individual cages with *ad libitum* access to water. Prior to the experiment, a cannula was placed in the cranial vena cava to minimize stress during blood sampling. The animals were administered with standard feed which was tested for the following mycotoxins: Aflatoxin, ochratoxin, ZEN, α-ZEL, and deoxynivalenol. Mycotoxin levels in the feed were estimated by common separation techniques with the use of immunoaffinity columns and HPLC (Hewlett Packard, type 1050 and 1100, Santa Clara, CA, USA) with fluorescent and/or UV detection techniques [34]. The obtained values were below the sensitivity of the tests.

#### 4.1.1. Experimental Design

The animals were divided into two experimental groups and a control group of six individuals each. Group EI gilts were orally administered 20 μg ZEN/kg BW (200% more than the highest NOAEL value) for 48 days, group EII gilts were orally administered 40 μg ZEN/kg BW (400% more than the highest NOAEL value) *per os* for 48 days, and group C animals were orally administered placebo for 48 days (Table 1). The placebo was a gelatine capsule filled with a control medium onto which ZEN was applied in experimental groups.

#### 4.1.2. Reagents

Sample weight of ZEN (Z-0167, Sigma Chemical Co., Steinheim, Germany) were administered *per os* daily in gelatine capsules before the morning feeding according to the weight of gilts. ZEN samples were diluted in 300 μL 96% ethyl alcohol (96% ethyl alcohol, SWW 2442-90, Polskie Odczynniki Chemiczne SA, Gliwice, Poland) to produce ZEN doses of 20 and 40 μg/kg BW. The resulting solutions were added to the feed, placed in gelatine capsules, and stored at room temperature for 12 h to evaporate the solvent.

### 4.2. Mycotoxicological Analyses

#### 4.2.1. Blood Sampling to Mycotoxicological Analyses

Blood was sampled on the first day of the experiment after the administration of ZEN, and ZON levels (average total ZEN and α-ZEL concentrations) were estimated. Blood samples for determinations of ZEN and α-ZEL concentrations were collected every 48 h, 1 h after mycotoxin administration. The samples were directly transferred to chilled centrifuge tubes containing heparin and were centrifuged at 3000 rpm for 20 min at the temperature of 4 °C. The resulting plasma was transferred to 3 mL polypropylene Eppendorf tubes 3810X, it was frozen and stored at −20 °C until analysis. Blood loss was compensated through the administration of multi-electrolyte solutions in amounts corresponding to the volume of collected samples.

#### 4.2.2. Analysis of ZEN and Its Metabolite in Blood Plasma

The presence of ZEN (see Figure 1) and α-ZEL (see Figure 1) in the blood plasma was determined by various separation methods with the use of immunoaffinity columns (Zearala-Test^TM^ Zearalenone Testing System, G1012, VICAM, Watertown, MA, USA) and HPLC with fluorescent detection.

#### 4.2.3. HPLC Analysis

The presence of ZEN and α-ZEL in blood plasma was determined using immunoaffinity columns (Zearala-TestTM Zearalenone Testing System, G1012, VICAM, Watertown, MA, USA) and Agilent 1260 series liquid chromatography (LC) coupled with a mass spectrometer (MS) (Agilent 6460, Agilent Technologies, Inc., Santa Clara, CA, USA). The prepared sample was identified with the use of a chromatographic column (Atlantis T3 3 μm 3.0 × 150 mm Column, Waters, AN Etten-Leur, Dublin, Ireland). The mobile phase consisted of 70% acetonitrile (LiChrosolvTM, No. 984 730 109, Merck-Hitachi, Mannheim, Germany), 20% methanol (LiChrosolvTM, No. 1.06 007, Merck-Hitachi, Mannheim, Germany), and 10% deionized water (Milipore Water Purification System, Millipore S.A. Molsheim, France) with an addition of 0.2% CH3 COOH. The flow rate was 0.4 mL/min., and the temperature of the oven column was 40 °C. The chromatographic analysis was completed in 4 min. Mycotoxin concentrations were determined according to the external standard and were expressed in ppb (ng/mL). The limits of detection (LODs) for individual mycotoxins were determined as the concentrations at which the signal-to-noise ratio decreased to three. The LOQ was estimated as the triple LOD value. Quantification limits (LOQs) were determined at 0.100 ng/mL for ZEN and α-ZEL. The correlation coefficient (r) for the calibration curve was 0.9996 and 0.9989 for ZEN and α-ZEL, respectively.

#### 4.2.4. Determination of Mycotoxin Bioavailability in Blood Plasma

The data were recorded and integrated with the use of the Computer Integrator POL-LAB application and CHROMAX for Windows v. 2000 software for processing chromatographic data (Pol-Lab Artur Dzieniszewski, Warszawa, Poland). Mycotoxin concentrations were determined according to the external standard method and given in terms of ng/mL.

### 4.3. Determination of Hormone (Estradiol and Testosterone) Concentrations in Blood Plasma

Blood was sampled every 48 h (25 times), 1 h after mycotoxin administration. The samples were transferred to chilled centrifuge tubes and were centrifuged at 3000 rpm for 20 min at the temperature of 4 °C. The resulting plasma was transferred to plastic polypropylene Eppendorf tubes, it was frozen and stored at −18 °C until analysis of selected steroid hormone levels.

Estradiol (see Figure 3) and T (see Figure 4) concentrations in the blood plasma were estimated by RIA procedures described for E_2_ by Hotchkiss et al. [43] and for T—by Kotwica and Williams [44]. Estradiol and T antibodies (obtained from the Institute of Animal Physiology, University of Warmia and Mazury in Olsztyn, Poland) were characterized by Szafrańska et al. [45]. The sensitivity of E_2_ and T assays was 5 pg and 2.5 ng (per tube), respectively. Intra-assay and inter-assay CVs for E_2_ and T were determined at 6.9% and 11.8% and 7.6% and 12.5%, respectively.

### 4.4. Bioavailability Factor

The daily dose of ZEN (20 or 40 µg/kg BW) was administered to each animal individually, which corresponds to exposure to 928.74–1856.8 µg ZEN/kg of a complete diet, depending on daily feed intake (Table 1).

The bioavailability factor (see Figure 5) was calculated as the ratio of ZEN + α-ZEL = ZOL bioavailability in peripheral blood (expressed as ppb) to ZEN concentration in the diet (expressed as ppb), expressed as the overall dose administered in each week of the experiment, according to a previously described method.

### 4.5. Statistical Analysis

Plasma concentrations of ZEN (see Figure 1), α-ZEL (see Figure 1), and ZON (see Figure 2) as well as E_2_ (see Figure 3) and T (see Figure 4) levels in the blood plasma of the examined gilts were presented as arithmetic means ($\bar{x}$) and SD (±). Statistical calculations were performed using the Statistica application (Statsoft Inc., Tulsa, OK, USA). Due to the applied ZEN dose and the length of application, arithmetic means were compared by one-way analysis of variance for systems with repeatable measurements. The homogeneity of variances in the compared groups (required in analyses of variance) was verified by the Brown–Forsythe test. Differences between groups were analyzed by Tukey’s honestly significant difference test (*p* < 0.05 or *p* < 0.01).

## Figures and Tables

**Table 1:** Daily feed intake in a restricted feeding regimen (kg/day) and mean dietary concentrations of ZEN per kg of feed (μg/kg feed).

Weeks of Exposure	Group EI			Group EII			Group C
	Feed Intake		Total ZEN Dose	Feed Intake		Total ZEN Dose	Feed Intake
	kg/day	µg/kg BW	µg/kg Feed	kg/day	µg/kg BW	µg/kg Feed	kg/day
I	1.7	664.4	390.82	1.75	1328.8	759.314	1.7
II	1.8	737.4	409.66	1.9	1474.8	776.210	1.85
III	1.9	819	431.05	1.9	1638	862.105	1.9
IV	2.0	912.2	456.1	1.9	1818.8	957.263	2.0
V	2.15	1014	471.62	2.2	2017.2	916.909	2.1
VI	2.1	1121.8	534.19	2.25	2243.6	997.155	2.2
VII	2.1	1232.4	586.85	2.2	2476.4	1125.636	2.2

## Abbreviations

ARs: Androgen receptors; BW: Body weight; C: Control group; CAS: Chemical Abstracts Service; EI: Experimental group I; EII: Experimental group II; E_2_: Estradiol; Eds: Endocrine disruptors; ERs: Estrogen receptors; ERα: Estrogen receptors alpha; ERβ: Estrogen receptors beta; HSDs: Hydroxysteroid dehydrogenases; LOD: Values below limits of detection; NOAEL: No-observed-adverse-effect level; PCNA: Proliferating cell nuclear antigen; SD: Standard deviation; Ts: Testosterone; ZEN: Zearalenone; α-ZEL: α-zearalenol; ZON: Total ZEN and α-ZEL.
